# A dermoid cyst fistulating with the transverse colon

**DOI:** 10.1308/003588412X13373405385935

**Published:** 2012-10

**Authors:** AZ Conway, T Hanna

**Affiliations:** ^1^Surrey and Sussex Healthcare NHS Trust,UK; ^2^North Devon Healthcare NHS Trust,UK

**Keywords:** Dermoid Cyst, Fistula, Colon, Transverse

## Abstract

We present a rare case of fistulation of a dermoid cyst with the transverse colon. We illustrate how an infected dermoid cyst can be diagnosed as an appendix abscess although the management of these is quite different. The general surgeon should be aware of this as a differential diagnosis for an appendix abscess.

Also known as dermoid cysts, mature cystic ovarian teratomas are benign tumours composed of two or more of the three germ layers (endoderm, mesoderm, ectoderm). They are the most common germ cell tumour and often asymptomatic although they may present with abdominal pain or an abdominal mass. The most common complication is torsion.^1^ However, they may also rupture, become infected, fistulate or undergo malignant transformation. While tumours invade the surrounding organs, particularly the rectum and sigmoid colon, invasion into the mucosa is not common and fistulation of a gynaecological tumour is rare.

Malignant and benign gynaecological tumours reported previously have been due to fistulation with the rectum, sigmoid colon or ileum. This is only the third case report of fistulation between a mature cystic ovarian teratoma and the transverse colon, and we give an illustration of the presentation and differential diagnosis, which is relevant to the general surgeon.

## Case history

A 26-year-old woman presented to a rural South African hospital with a 1-day history of abdominal pain associated with nausea. On examination she appeared unwell, had cardiovascular compromise with a tachycardia of 120 beats per minute and blood pressure of 80/52mmHg, and remained afebrile. She had signs of localised peritonitis in the right iliac fossa. Digital rectal examination was unremarkable. Vaginal examination revealed a palpably abnormal cervix and bright red blood although she was not currently menstruating. A urine dipstick was negative for infection and β-human chorionic gonadotrophin. She had presented with similar symptoms one year previously with a suspected appendix abscess. This was treated with a percutaneous drain and antibiotics, and her symptoms resolved. She had a past medical history of abdominal tuberculosis and was HIV positive, currently on antiretroviral treatment with a CD4 count of 254/µl.

**Figure 1:**
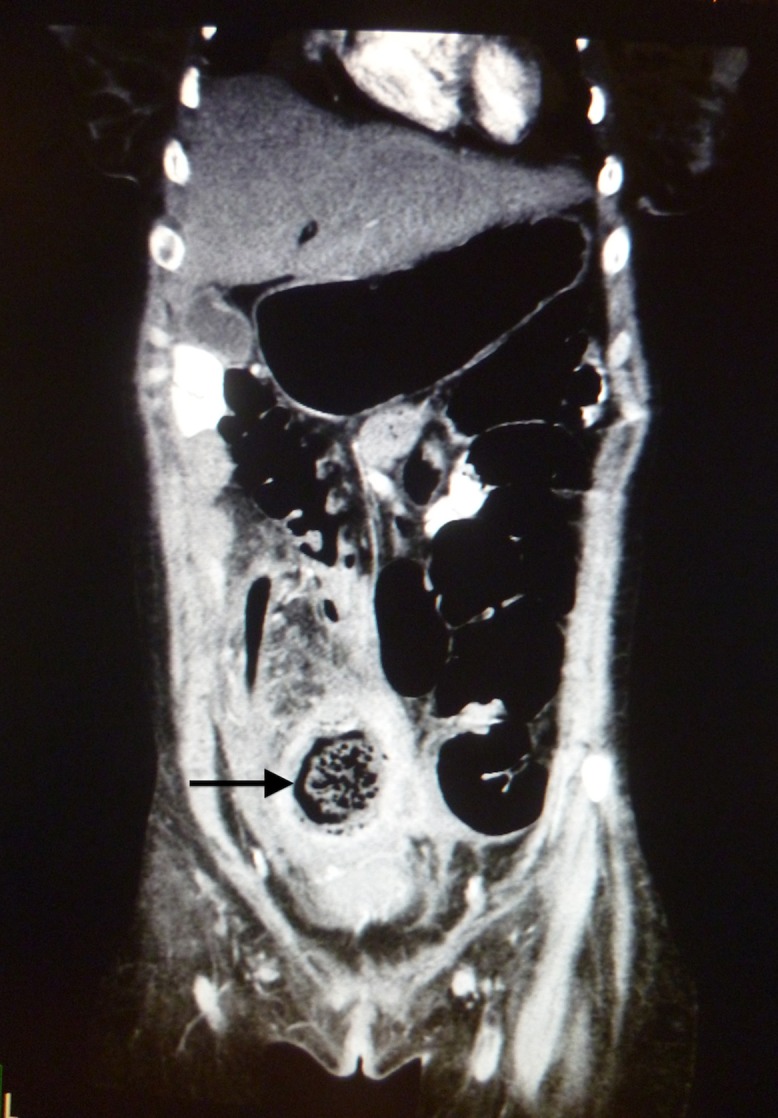
Coronal computed tomography illustrating the inflammatory mass in the pelvis and right iliac fossa

**Figure 2:**
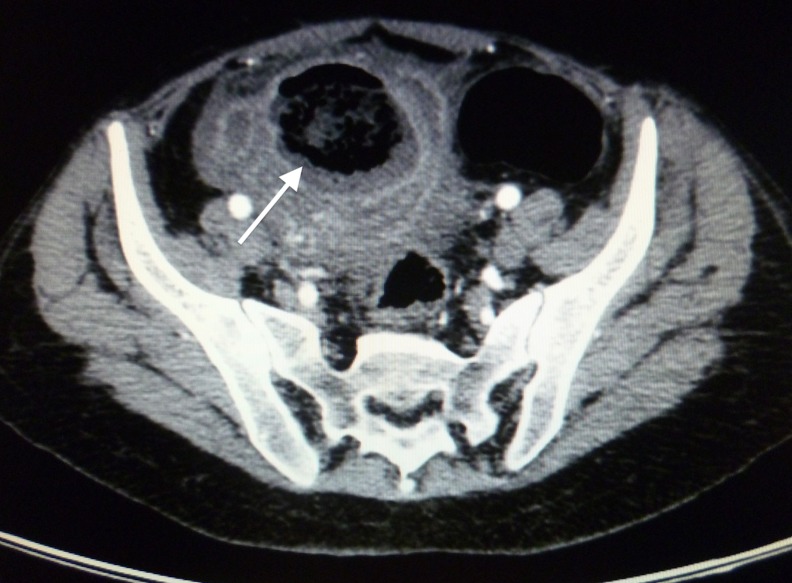
Transverse computed tomography illustrating the inflammatory mass

In view of the patient’s history and concurrent medical problems, computed tomography (CT) was performed. This revealed a multiloculated abscess in the pelvis with a calculus noted adjacent to the collections (Figs 1 and 2). The differential diagnoses from the CT included a ruptured appendix or a ruptured tubo-ovarian abscess.

The previous intra-abdominal abscess and abdominal tuberculosis may mean that an abdominal operation would be challenging technically. The patient would also be more susceptible to post-operative infection and poor healing due to her immunodeficiency although a CD4 count of over 250/µl is considered adequate for an elective operation at this hospital, which may have been why percutaneous drainage was performed previously. With clinical evidence of sepsis, local peritonism on abdominal examination and radiological evidence of a probable ruptured viscus, we proceeded to laparotomy.

Intra-operative findings revealed a mass lying superior to the uterus fistulating with the transverse colon. There was some free turbid fluid in the abdomen but minimal adhesions from the patient’s previous abdominal tuberculosis. The mass was excised and, on opening it, contents consistent with a teratoma were found (hair and sebaceous fluid) as well as a flat worm, most likely an incidental finding. The fistulous tract entering the transverse colon was excised and the defect closed primarily.

The patient completed a 7-day course of antibiotics, made an uneventful recovery and was discharged home on post-operative day 14. The histology confirmed a mature cystic ovarian teratoma. She was followed-up at six weeks and was doing well.

## Discussion

Mature cystic ovarian teratomas are the most common germ cell neoplasm and have been identified intra-operatively as the most common ovarian tumour to be excised in some series.^2^ Many remain asymptomatic while some patients suffer from abdominal pain or non-specific symptoms.^3^ These slow growing tumours can be managed non-surgically if small in size and asymptomatic although, if removal is required, a cystectomy sparing the ovary can be performed laparoscopically. The surgeon must be careful to avoid leakage of the contents as this may lead to a chemical peritonitis.^4^ Malignant degeneration occurs rarely, recent studies identifying only 1 in 517 cases.^3^

Torsion of a teratoma is the most significant cause of morbidity. Torsion may be associated with rupture of the cyst and also fistulation. It is thought that if torsion is complete, this will interrupt the arterial blood supply, causing necrosis and therefore rupture. However, if the torsion does not occlude the arterial blood supply completely, the cyst wall may undergo an inflammatory response, leading to adhesions with nearby structures. The adhered area may continue gradually with an inflammatory response and necrosis, eventually leading to fistulation with the adjacent structure^1^ or, alternatively, infection or direct invasion due to malignant change.

Interestingly, a case of squamous cell carcinoma arising from a teratoma of the uterine cervix in a patient with HIV has been reported.^5^ Although a diagnosis of cervical cancer had not been made in this patient, a palpably abnormal cervix in the presence of HIV is suspicious as cervical cancer is an AIDS defining illness. Following recovery from the operation for removal of the teratoma and closure of the fistula of the transverse colon, the patient was referred to the gynaecologists who were based in a separate hospital.

## Conclusions

The differential diagnoses for an infected teratoma include an appendix abscess and, despite imaging, the diagnosis may be made intra-operatively. The management of both is quite different. In our case, at the first presentation the patient was managed as having an appendix abscess, which may have led to the fistulation due to the percutaneous drainage of the abscess at her first presentation. Due to her immunocompromised status, a conservative approach would have been the preferred option. Teratomas can fistulate with the transverse colon as well as other sections of the large or small bowel and, although rare, may be due to malignancy, which must therefore be identified and managed appropriately.
